# Supplementary material for: CA3 hippocampal synaptic plasticity supports ripple physiology during memory consolidation
Source: Nat Commun. 2023 Dec 14;14:8312. doi: 10.1038/s41467-023-42969-x (PMC10721822; doi:10.1038/s41467-023-42969-x)
Supplement: Supplementary file 1 — Supplementary Information [file 41467_2023_42969_MOESM1_ESM.pdf]

Supplementary Material

Title

CA3 hippocampal synaptic plasticity supports ripple physiology during memory consolidation.

Author list

Hajer El Oussini[1#], Chun-Lei Zhang[1#3] Urielle François[1#], Cecilia Castelli[1], Aurélie Lampin-Saint-Amaux[1], Marilyn Lepleux[1], Pablo Molle[1], Legeolas Velez[1], Cyril Dejean[2], Frédéric Lanore[1], Cyril Herry[2], Daniel Choquet[1#] and Yann Humeau[1#□].

Affiliations

[1] : Univ. Bordeaux, CNRS, IINS, UMR 5297, F-33000 Bordeaux, France

[2] : Univ. Bordeaux, INSERM, Neurocentre Magendie, U1215, F-33000 Bordeaux, France

[3] : Present address : Sorbonne Université, CNRS, INSERM, Institut de Biologie Paris Seine (IBPS), Neurosciences Paris Seine (NPS), Team Synaptic Plasticity and Neural Networks, F-75005 Paris, France

[#]: Contributed equally to the work.

[□]: Corresponding author : yann.humeau@u-bordeaux.fr

Inventory of Supporting Information

This word document includes 6 supplementary materials:

Supplementary figure 1 + legend

Supplementary figure 2 + legend

Supplementary figure 3 + legend

Supplementary figure 4 + legend

Supplementary figure 5 + legend

Supplementary Table for statistics

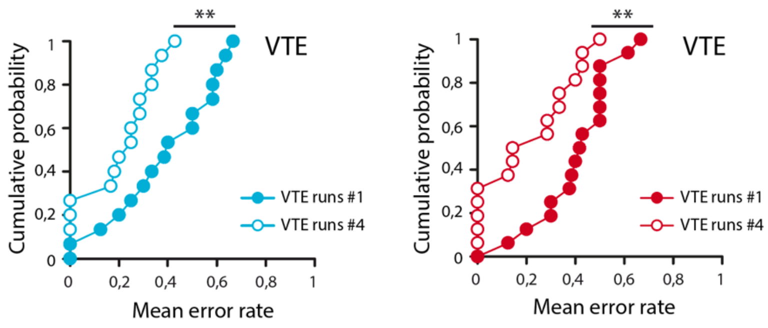

Sup. Figure 1: dHPC AMPAR X-linking preserve DSA rule encoding.
Same presentation as Figure 2. Cumulative single animal data for choice accuracy at VTE runs in session#1 and session#4 after pre-learning FaB- (blue, Left, n=16 independent animals) or IgG- (Red, right, n=17 independent animals) injections. t-tests were used. **: p<0.01.

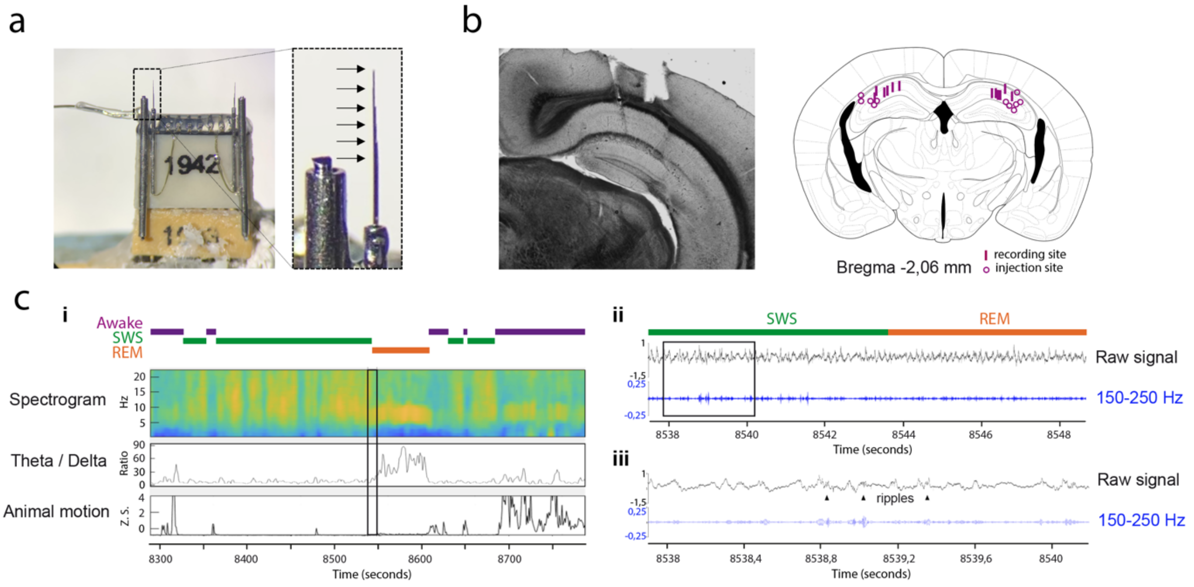

Sup. Figure 2: Combined dHPC electrophysiological and pharmacological experiments.
a: photograph of the design implant. Ripple recordings are performed *via* 2x6 wires cut at 200 micrometres intervals in order to target the stratum pyramidale of the CA3 region. Lateral to each bundle, are positioned cannulas that will be used for drug injection. **b**: micrograph of coronal slice of implanted mouse brain. Arrows identified the bundle of recording wires, and the tip of the implanted cannula. To perform injection, an injection cannula projects out of 1 millimetre within the hippocampus and is retracted back during the injection in order to cover a large portion of the dHPC. Template drawing is from “the mouse brain” Paxinos and Franklin. **c**: Analytical pipeline for sleep and ripple detection. i) Animal tracking (motion) allows separating awake and sleeping states. Within sleep periods, LFP analysis of the Theta/Delta range separate REM (high theta/low delta) and SWS (low theta/high delta) phases. ii): In parallel, LFP are band pass filtered between 150-250 Hz in order to extract ripples oscillations (see methods). iii) A zoom on detected ripples is shown (same period as black square inset in ii).

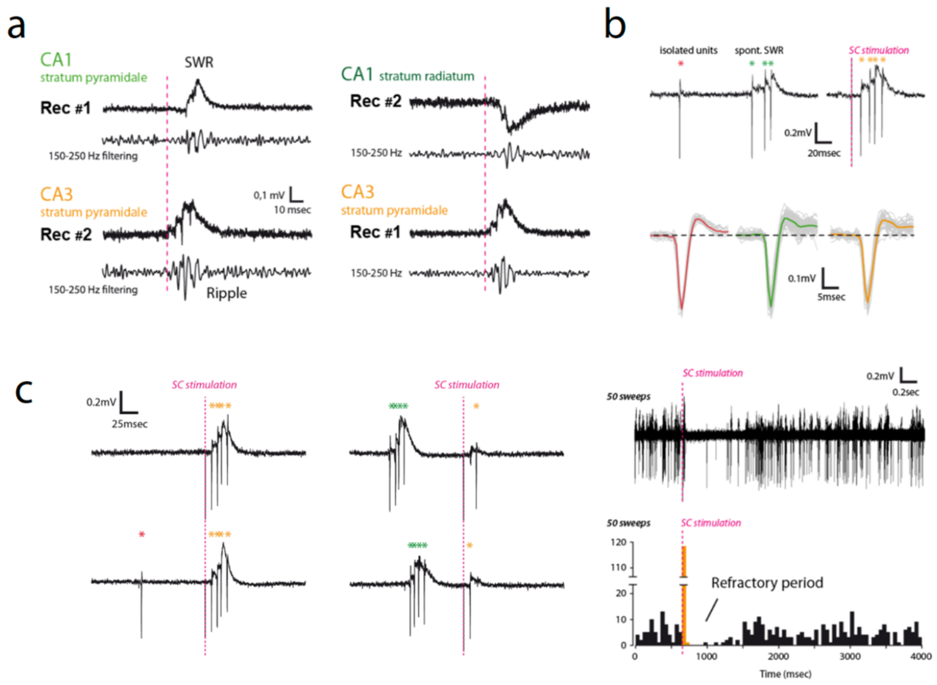

Sup. Figure 3: Characteristics of SWRs recorded in hippocampal acute slices.
a: SPW-Rs events recorded in acute hippocampal slice are composed of dendritic synaptic wave that polarity is changed depending on electrode position (upward in CA1-sp (left), downward in CA1-sr (right)). Filtering at 150-250 Hz shows high frequency oscillations (ripples) associated to the wave. Note that a significant delay exists between CA3 SWRs and those recorded in CA1 area, suggesting that they are occurring first in the CA3 region.
b: Typical example of a CA3-sp single unit recording showing that spontaneous or evoked (through CA3 axons stimulations) SWRs are indeed leading to focal neuronal activation. Bottom: waveforms of the single unit in the three conditions (alone, spontaneous SWRs and evoked SWRs). **c**: interaction between spontaneous and evoked SWRs. Typical example of refractory period observed for consecutive SWRs that probably limit their activity in acute slices. Left: examples of collision between spontaneous and evoked SWRs. Right: No spontaneous SWR is observed after the generation of an evoked SWR, suggesting that these oscillations may require time-dependent regenerative mechanisms.

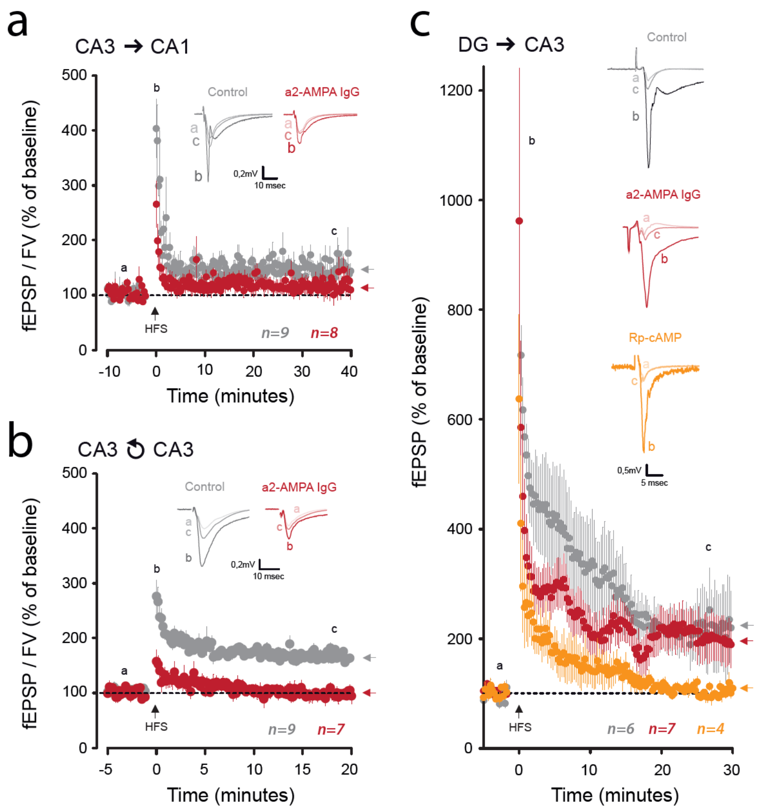

Sup. Figure 4: AMPAR X-linking impair LTP at CA3□CA1, CA3□CA3 but not DG□CA3 synapses.

a: Time course of fEPSP/FV slopes ratio before and after high frequency stimulations (HFS) in control (grey) and AMPAR X-linking conditions (red). Note the absence of potentiation in presence of anti GluA2 IgGs (arrows). In insert, representative traces obtained during baseline (a), immediately after HFS (b) and 35 minutes after HFS (c) in both conditions. **b**: Same presentation as in **a**. HFS stimulation was applied within the CA3 *stratum radiatum* to isolate CA3□CA3 recurrent synapses. Note the absence of potentiation in presence of anti GluA2 IgGs (arrows). In insert, representative traces obtained during baseline (a), immediately after HFS (b) and 20 minutes after HFS (c) in both conditions. **c**: Same presentation as in **a**. HFS stimulation was applied within the CA3 *stratum lucidum* to isolate DG□CA3 synapses. Note the potentiation that is still present in the anti GluA2 IgGs condition, but not when slice was preincubated with Rp-cAMP to block PKA activity (arrows). In insert, representative traces obtained during baseline (a), immediately after HFS (b) and 20 minutes after HFS (c) in all conditions. The number of independent experiments is indicated.

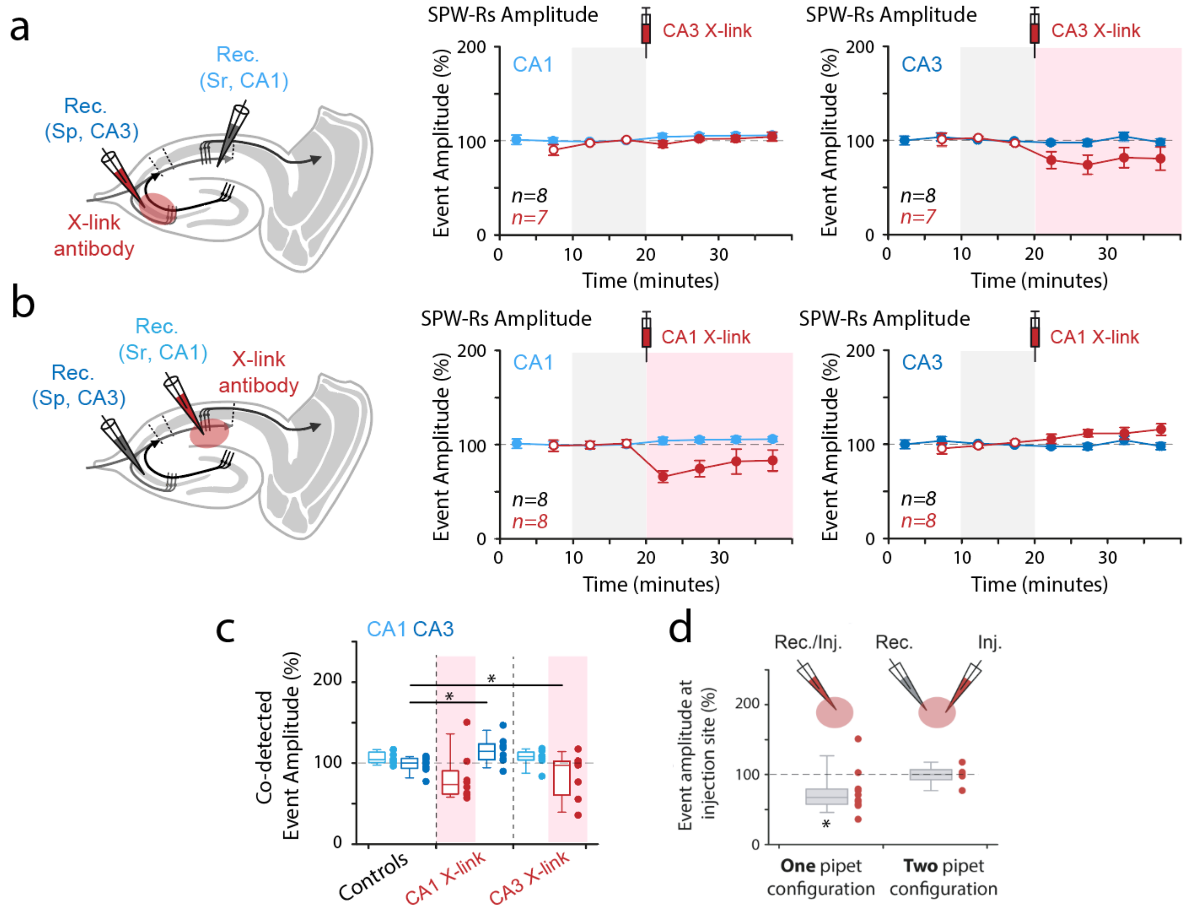

Sup. Figure 5: AMPAR X-linking effect on SWR amplitude is due to injection procedure.
a-b: Effect of AMPAR X-linking on spontaneous SPWRs amplitude was tested in *in situ* preparations by pressure injection of anti-GluA2 IgGs. Left: schematic of the experiment. Right: Time course of SPW-Rs amplitude in CA1 and CA3 regions. The “light red” area indicate that events are recorded in the IgG-injected area. All except red colour indicate time course of the same parameter in control conditions. Open red circles are the IgG preparations before the injections. **c**: All single experiments and average values after 20 minutes, in control or after IgG injections. Note that a significant decrease in SPW-R amplitude is observed at the locus of IgG injection. **d:** We compared the local effects of anti-GluA2 antibodies on SWR amplitude in experiments in which it was introduced via the recording pipet (One pipet configuration) or using another pipet than the recording one (two pipet configuration). As can be seen, the decrease in amplitude of SWRs was due to the positive pressure applied in the pipet that probably moved away the tissue locally. Thus we conclude that, as previously observed[15] that AMPAR X-linking was not affecting basal transmission, and thus leave spontaneous SWRs unaffected. t-test were used. In case that sample distribution was not normal – after Shapiro-Wilk test – a Mann-Whitney Rank Sum test was used. *p<0.05. The number of independent experiments is indicated.

Sup. Table: statistical tests.

Figure 1	Panel	condition	Test	stats					
	d	pre learning	two way anova	session	Comparison	Diff of Means	t	P	
				1	FAB vs. IGG	0,0496	0,368	0,713	
				2	IGG vs. FAB	0,0289	0,214	0,83	
				3	IGG vs. FAB	0,138	1,021	0,308	
				4	IGG vs. FAB	0,14	1,043	0,298	
				5	IGG vs. FAB	0,581	4,315	<0,001	***
				6	IGG vs. FAB	0,544	3,973	<0,001	***
				7	IGG vs. FAB	0,272	2,019	0,044	**
				8	IGG vs. FAB	0,254	1,887	0,06	
				9	IGG vs. FAB	0,253	1,881	0,061	
				10	IGG vs. FAB	0,173	1,266	0,207	
	d	Pre rest	two way anova	session	Comparison	Diff of Means	t	P	
				1	Fab vs. IgG	0,128	0,744	0,458	
				2	IgG vs. Fab	0,0306	0,177	0,86	
				3	IgG vs. Fab	0,322	1,868	0,064	
				4	Fab vs. IgG	0,0756	0,438	0,662	
				5	IgG vs. Fab	0,538	3,118	0,002	**
				6	IgG vs. Fab	0,426	2,47	0,015	*
				7	IgG vs. Fab	0,322	1,865	0,064	
				8	IgG vs. Fab	0,244	1,414	0,16	
				9	IgG vs. Fab	0,136	0,786	0,433	
				10	IgG vs. Fab	0,172	0,998	0,32	
	d	pre test	two way anova	session	Comparison	Diff of Means	t	P	
				1	IGG vs. FAB	0,0529	0,41	0,683	
				2	FAB vs. IGG	0,0667	0,517	0,606	
				3	IGG vs. FAB	0,005	0,0388	0,969	
				4	FAB vs. IGG	0,104	0,807	0,422	
				5	FAB vs. IGG	0,259	2,01	0,047	*
				6	IGG vs. FAB	0,239	1,85	0,067	
				7	IGG vs. FAB	0,138	1,069	0,287	
				8	FAB vs. IGG	0,266	2,066	0,041	*
				9	FAB vs. IGG	0,18	1,396	0,166	
				10	FAB vs. IGG	0,089	0,69	0,491	
	e	encoding FAB	Paired t-test					<0,001	***
		encoding IGG	Paired t-test					0,006	**
		Consol.FAB	Paired t-test					0,784	
		Consol. IGG	Paired t-test					<0,001	***
	f	Consol. FAB	Paired t-test					0,378	
		Consol.IGG	Paired t-test					0,016	*
	g	Consol. FAB	Paired t-test					0,964	
		Consol. IGG	Paired t-test					0,250	
**Figure 2**	**Panel**	**condition**	**Test**	**stats**					
	a	VTE no VTE	Mann-Whitney					0,002	**
	c	FAB noVTE #1 & #5	t-test					0,005	**
		IGG noVTE #1 &#5	t-test					0,862	
	d	VTE errors	two way anova	session	Comparison	Diff of Means	t	P	
				1	IGG vs. FAB	0,0159	0,23	0,818	
				2	IGG vs. FAB	0,108	1,536	0,126	
				3	IGG vs. FAB	0,0986	1,427	0,155	
				4	IGG vs. FAB	0,00722	0,104	0,917	
				5	IGG vs. FAB	0,276	3,934	<0,001	***
				6	IGG vs. FAB	0,216	3,012	0,003	**
				7	IGG vs. FAB	0,195	2,786	0,006	**
				8	IGG vs. FAB	0,093	1,303	0,194	
				9	IGG vs. FAB	0,099	1,366	0,173	
				10	IGG vs. FAB	0,125	1,641	0,102	
	d	no VTE errors	two way anova	session					

				1	FAB vs. IGG	0,0805	1,113	0,267	
				2	FAB vs. IGG	0,094	1,3	0,195	
				3	FAB vs. IGG	0,0182	0,252	0,801	
				4	IGG vs. FAB	0,0578	0,771	0,442	
				5	IGG vs. FAB	0,197	2,682	0,008	**
				6	IGG vs. FAB	0,0541	0,735	0,463	
				7	FAB vs. IGG	0,0188	0,26	0,795	
				8	IGG vs. FAB	0,046	0,625	0,533	
				9	IGG vs. FAB	0,0129	0,178	0,859	
				10	IGG vs. FAB	0,075	1,019	0,309	
	e	no VTE FAB	Mann-Whitney					<0,001	***
		VTE FAB	Mann-Whitney					0,003	**
		no VTE IGG	t-test					0,748	
		VTE IGG	Mann-Whitney					0,724	
**Sup Figure 1**	Panel	condition	Test	stats					
		FAB VTE #1 & #4	t-test					0,009	**
		IGG VTE #1 & #4	t-test					0,003	**
**Figure 3**	Panel	condition	Test	stats					
	c	frequency	Wilcoxon					0,497	
		amplitude	Paired t-test					0,368	
	d	frequency	Paired t-test					0,235	
		amplitude	Wilcoxon					0,570	
	e	frequency	Paired t-test					0,029	*
		amplitude	Paired t-test					0,024	*
	f	frequency	Wilcoxon					0,313	
		amplitude	Paired t-test					0,265	
**Figure 4**	Panel	measure	Test	stats					
	h	CA3 IGG & Ctl Hz	t-test					0,756	
		CA1 IGG & Ctl Hz	t-test					0,803	
**Sup Figure 5**	Panel	measure	Test	stats					
	c	CA1 EPSP ampl CA1 IGG VS Ctrl	t-test					0,225	
		CA3 EPSP ampl CA1 IGG VS Ctrl	t-test					0,024	*
		CA1 EPSP ampl CA3 IGG VS Ctrl	t-test					0,962	
		CA3 EPSP ampl CA3 IGG VS Ctrl	Mann Whitney					0,028	*
	d	ampl. SPWR 1pip VS before	Mann Whitney					0,038	*
		ampl. SPWR 2pip VS before	Mann Whitney					0,534	
**Figure 5**	Panel	condition	Test	stats					
	c	EPSP slope early	wilcoxon					0,031	*
		EPSP slope late	paired t-test					0,012	*
		SPWR freq early	wilcoxon					0,310	
		SPWR freq late	wilcoxon					0,020	*
	e	EPSP slope early IGG	paired t-test					0,007	**
		EPSP slope late IGG	paired t-test					0,555	
		EPSP slope early IGG/Ctrl	Mann-Whitney					<0,001	***
		EPSP slope late IGG/ctrl	t-test					0,004	**
		SPWR freq early IGG	paired t-test					0,061	
		SPWR freq late IGG	paired t-test					0,335	
		SPWR freq early IGG/Ctrl	Mann-Whitney					0,02	*
		SPWR freq late IGG/Ctrl	Mann-Whitney					0,138	
**Figure 6**	Panel	condition	Test	stats					
	d	controls	paired t-test					<0,001	***
	d	BIRA+NA	paired t-test					0,501	
	e	controls	t-test					0,008	**
	e	BIRA+NA	t-test					0,635	
**Figure 7**	Panel	condition	Test	stats					
	b	controls	paired t-test					0,571	
	b	BIRA+NA	paired t-test					0,022	*
