# Supplementary material for: CA3 hippocampal synaptic plasticity supports ripple physiology during memory consolidation
Source: Nat Commun. 2023 Dec 14;14:8312. doi: 10.1038/s41467-023-42969-x (PMC10721822; doi:10.1038/s41467-023-42969-x)
Supplement: Supplementary file 3 — Reporting Summary [file 41467_2023_42969_MOESM3_ESM.pdf]

nature portfolio

Corresponding author(s): Yann Humeau

Last updated by author(s): Sep 27, 2023

Reporting Summary

Nature Portfolio wishes to improve the reproducibility of the work that we publish. This form provides structure for consistency and transparency in reporting. For further information on Nature Portfolio policies, see our Editorial Policies and the Editorial Policy Checklist.

Statistics

For all statistical analyses, confirm that the following items are present in the figure legend, table legend, main text, or Methods section.

n/a	Confirmed	
☐	☒	The exact sample size (*n*) for each experimental group/condition, given as a discrete number and unit of measurement
☐	☒	A statement on whether measurements were taken from distinct samples or whether the same sample was measured repeatedly
☐	☒	The statistical test(s) used AND whether they are one- or two-sided<br>*Only common tests should be described solely by name; describe more complex techniques in the Methods section.*
☐	☒	A description of all covariates tested
☐	☒	A description of any assumptions or corrections, such as tests of normality and adjustment for multiple comparisons
☐	☒	A full description of the statistical parameters including central tendency (e.g. means) or other basic estimates (e.g. regression coefficient) AND variation (e.g. standard deviation) or associated estimates of uncertainty (e.g. confidence intervals)
☐	☒	For null hypothesis testing, the test statistic (e.g. *F*, *t*, *r*) with confidence intervals, effect sizes, degrees of freedom and *P* value noted<br>*Give P values as exact values whenever suitable.*
☒	☐	For Bayesian analysis, information on the choice of priors and Markov chain Monte Carlo settings
☒	☐	For hierarchical and complex designs, identification of the appropriate level for tests and full reporting of outcomes
☒	☐	Estimates of effect sizes (e.g. Cohen's *d*, Pearson's *r*), indicating how they were calculated

Our web collection on statistics for biologists contains articles on many of the points above.

Software and code

Policy information about availability of computer code

Data collection	The scripts that have been used for ripple analysis can be found at the following address: https://figshare.com/account/home#/projects/179595.
Data analysis	For data analysis, statistical comparisons and figure production, the sigmaplot 12 suite from systatsoftware was used.

For manuscripts utilizing custom algorithms or software that are central to the research but not yet described in published literature, software must be made available to editors and reviewers. We strongly encourage code deposition in a community repository (e.g. GitHub). See the Nature Portfolio guidelines for submitting code & software for further information.

Data

Policy information about availability of data

All manuscripts must include a data availability statement. This statement should provide the following information, where applicable:

- Accession codes, unique identifiers, or web links for publicly available datasets
- A description of any restrictions on data availability
- For clinical datasets or third party data, please ensure that the statement adheres to our policy

All single data can be found at the following address: https://figshare.com/account/home#/projects/179595

Research involving human participants, their data, or biological material

Policy information about studies with human participants or human data. See also policy information about sex, gender (identity/presentation), and sexual orientation and race, ethnicity and racism.

Reporting on sex and gender	N/A
Reporting on race, ethnicity, or other socially relevant groupings	N/A
Population characteristics	N/A
Recruitment	N/A
Ethics oversight	N/A

Note that full information on the approval of the study protocol must also be provided in the manuscript.

Field-specific reporting

Please select the one below that is the best fit for your research. If you are not sure, read the appropriate sections before making your selection.

☒ Life sciences ☐ Behavioural & social sciences ☐ Ecological, evolutionary & environmental sciences

For a reference copy of the document with all sections, see nature.com/documents/nr-reporting-summary-flat.pdf

Life sciences study design

All studies must disclose on these points even when the disclosure is negative.

Sample size	The specific sample size for each analysis is summarised in the figure legends, main text and methods. Throughout the manuscript, n refers to the number of independent experiments or to the number of animals as indicated for each analysis (see legends). For behavioral experiments, a minimal sample size of 5 animais was chosen to ensure the findings were reproducible, while minimising animal numbers.
Data exclusions	For in vivo pharmacological experiments, animals were excluded if histological controls indicated off-target implantations, or if bilateral injections were not completed. For pharmacological effects on ripples during sleep, experiments were considered if a good injection was done in the same side as the used electrophysiological channel. A few excluded experiment are listed in the "source data file" available online https://figshare.com/account/home#/projects/179595.
Replication	Key experiments (pre-rest or pre-learning AM PAR X-linking using two different strategies) were performed on at least 3 different cohorts of Wf or KI mice, and gave similar results. In vivo pharmacological controls were contributed by injection failures and eventual off targets, but always included significant groups of yet published controls.
Randomization	All cohorts were randomized, including control and X-linking conditions, and main results were repeated several times.
Blinding	Ali analyses were done blind to the presence or absence of the AMPAR X-linking (either animal genotype or pharmacological treatment).

Reporting for specific materials, systems and methods

We require information from authors about some types of materials, experimental systems and methods used in many studies. Here, indicate whether each material, system or method listed is relevant to your study. If you are not sure if a list item applies to your research, read the appropriate section before selecting a response.

Materials & experimental systems

n/a	Involved in the study	
☐	☒	Antibodies
☒	☐	Eukaryotic cell lines
☒	☐	Palaeontology and archaeology
☐	☒	Animals and other organisms
☒	☐	Clinical data
☒	☐	Dual use research of concern
☒	☐	Plants

Methods

n/a	Involved in the study	
☒	☐	ChIP-seq
☒	☐	Flow cytometry
☒	☐	MRI-based neuroimaging

Antibodies

Antibodies used	Monoclonal whole lgGl-K and Fab fragments recognising the extracellular domain of GluA2 (clones 15Fl and 14B11, gifts from E. Gouaux), were prepared using the purified GluA2 receptor in detergent solution as the antigen.
Validation	Validation of used antibodies was done in Penn et al., 2017. (10.1038/nature23658)

Animals and other research organisms

Policy information about studies involving animals; ARRIVE guidelines recommended for reporting animal research, and Sex and Gender in Research

Laboratory animals	Experiments in this manuscript were conducted on 6 to 12 weeks old male mice belonging to two strains: C57BL6/J wild type and C57BL6/J transgenic AP-GluA2 knock-In (KI, maintained on a C57BL6/J background) strains. Mice were kept on a 12-hour light/dark cycle and provided with ad libitum food and water, except for food restriction associated with behavioural testing (see below). Mice were housed with 3-5 littermates except when demanded by the protocol.
Wild animals	No wild animais were used in this study.
Reporting on sex	Only male mice were used.
Field-collected samples	No field collected animais were used in this study.
Ethics oversight	All procedures were validated by the ethical committee of animal experimental of Bordeaux Universities and the French ministry of Agriculture (CE50; Animal Facility PIV-EXP, APAFIS#18507-201901118522837; Animal Facility A1, APAFIS#4552 2016031019009163; Animal Facilities Neurocentre Magendie and PIV-EXPE, APAFIS#13515- 2018021314415739).

Note that full information on the approval of the study protocol must also be provided in the manuscript.
